# DPCrossU-Net: a dual-branch parallel CNN–Transformer network for lung nodule segmentation

**DOI:** 10.3389/fonc.2026.1802047

**Published:** 2026-06-09

**Authors:** Xiya Guan, Wen Zhu, Fangxiang Wu

**Affiliations:** 1 School of Mathematics and Statistics, Hainan Normal University, Haikou, China; 2 School of Mathematics and Systems Science Guangdong Polytechnic Normal University, Guangzhou, China; 3 Division of Biomedical Engineering and Department of Mechanical Engineering, University of Saskatchewan, Saskatoon, SK, Canada

**Keywords:** CNN–Transformer hybrid model, dual-branch architecture, feature fusion, LIDC-IDRI, lung nodule segmentation

## Abstract

**Introduction:**

Accurate segmentation of lung nodules in CT images is essential for early lung cancer screening and computer-aided diagnosis, yet remains challenging due to small target size, complex boundaries, and the limitations of existing convolutional or Transformer-based architectures in balancing local detail and global context modeling.

**Methods:**

We propose DPCrossU-Net, a dual-branch parallel encoder–decoder network that integrates convolutional and Vision Transformer representations. The encoder employs parallel CNN and ViT branches with a Cross-Attentive Fusion (CAF) module to adaptively combine local texture and global semantic features. Multi-scale atrous convolutions are introduced at the bottleneck to enhance sensitivity to small nodules, while a dual-branch Detail Context Fusion (DCF) block in the decoder improves boundary reconstruction.

**Results:**

Experiments conducted on the public LIDC-IDRI dataset demonstrate that DPCrossU-Net achieves a Dice score of 85.89%, outperforming the baseline U-Net and showing superior performance, particularly in small-nodule and complex-background scenarios.

**Discussion:**

These results indicate that synergistically combining parallel CNN–Transformer feature extraction with adaptive cross-branch fusion effectively enhances lung nodule segmentation. DPCrossU-Net provides a robust and clinically applicable solution, offering improved accuracy for early lung cancer analysis and potential support for future intelligent diagnostic systems.

## 1 Introduction

Lung cancer (1) frequently manifests as lung nodules in computed tomography (CT) images (2) at an early stage, and accurate segmentation of nodule regions is a prerequisite for subsequent benign–malignant assessment and pathological subtype determination. Although chest CT provides high spatial resolution and rich diagnostic information, the continuous growth in examination demand has resulted in a substantial increase in scan data per patient, rendering manual image interpretation increasingly time-consuming. Moreover, lung nodules exhibit pronounced variability in size, shape, and imaging characteristics—such as ground-glass opacity, calcification, and lobulation—which requires radiologists to examine a large number of slices sequentially to ensure diagnostic consistency, thereby imposing a considerable clinical burden. To improve diagnostic efficiency and consistency, computer-aided diagnosis (CAD) systems (3) have been increasingly adopted in clinical practice. Nonetheless, the expanding scale of CT data and the growing demand for intelligent diagnosis place higher requirements on the accuracy, robustness, and automation level of CAD systems. In this context, automated analysis of CT images has become particularly critical. Therefore, developing an automatic and reliable lung nodule segmentation technique (4) remains essential for improving clinical screening efficiency and facilitating early lung cancer detection.

In the early stages of automated lung nodule segmentation, traditional machine learning–based methods (5) were widely applied. These approaches typically relied on handcrafted features—such as grayscale distributions, shape descriptors, texture statistics, and filter-based local responses (6)—in combination with classical segmentation algorithms, including thresholding (7), region growing (8), and edge detection (9). Although such methods demonstrated practical effectiveness under constrained conditions, they are highly dependent on feature engineering and lack sufficient flexibility to capture the complex and heterogeneous imaging characteristics of lung nodules. This limitation becomes particularly evident in cases involving ground-glass opacity, blurred boundaries, or nodules adjacent to vessels and the chest wall. Consequently, their segmentation performance often fails to meet the stringent accuracy and robustness requirements of clinical imaging analysis.

With the rapid advancement of deep learning, end-to-end segmentation models based on convolutional neural networks (CNNs) (10) have become the mainstream approach for lung nodule segmentation. U-Net and its variants, originally proposed by Ronneberger et al. (11), have demonstrated strong performance by leveraging multi-scale feature extraction and skip connections to fuse low-level spatial information with high-level semantic features. Oktay et al. (12) proposed Attention U-Net, which introduces attention gates into the U-Net architecture to selectively emphasize salient regions and suppress irrelevant background responses, thereby improving medical image segmentation performance. ResUNet++ was subsequently developed by Jha et al. (13) by incorporating residual learning, multi-scale feature extraction, and squeeze-and-excitation mechanisms. Nonetheless, the inherently local receptive fields of convolutional operations limit the ability of CNN-based models to capture long-range dependencies and global contextual information, which are critical for accurately segmenting nodules with complex morphology and ambiguous boundaries. To mitigate this limitation, Transformer-based architectures (14) have been introduced into medical image analysis owing to their powerful self-attention mechanisms for global dependency modeling. MedT, proposed by Valanarasu et al. (15), employs gated axial attention to enhance contextual representation while reducing computational overhead, and has shown improved performance across multiple medical segmentation tasks. TransFuse, introduced by Zhang et al. (16), further explores CNN–Transformer fusion through parallel convolutional and Transformer streams, enabling complementary local–global feature learning. Despite these advances, Transformer-based models often exhibit limited capability in preserving fine-grained spatial details and face challenges in effective multi-scale feature fusion, particularly when applied to small targets such as lung nodules. Consequently, how to effectively integrate the local texture modeling strengths of CNNs with the global contextual awareness of Transformers within a unified and collaborative framework remains an open and critical research problem.

In light of these challenges, lung nodule segmentation requires a hybrid framework capable of jointly modeling fine-grained local appearance and long-range contextual information while maintaining effective coordination across different feature representations. To address this need, we propose DPCrossU-Net, a dual-branch parallel encoder–decoder segmentation network tailored for complex lung nodule scenarios. Unlike conventional hybrid models that rely on simple feature concatenation or sequential fusion, DPCrossU-Net is designed to promote complementary representation learning and structured collaboration between heterogeneous feature branches. This design provides three key advantages: (1) it enables simultaneous preservation of detailed spatial cues and holistic semantic context, reducing the trade-off between boundary accuracy and global consistency; (2) it facilitates coherent information exchange across feature representations, improving robustness to variations in nodule size, shape, and appearance; and (3) it enhances structural reliability during feature reconstruction, leading to more stable boundary delineation in challenging anatomical regions.

Owing to these properties, DPCrossU-Net achieves a balanced integration of local detail perception and global semantic understanding, making it well suited for accurate and reliable lung nodule segmentation. The main contributions of this work are summarized as follows:

1. We propose DPCrossU-Net, a dual-branch parallel encoder that integrates multi-scale convolutional and Transformer-based representations. Parallel CNN and ViT branches are employed to capture fine-grained spatial textures and long-range dependencies, respectively, enabling complementary modeling of lung nodule features.
2. To alleviate inherent discrepancies between CNN and ViT feature representations, a Cross-Attention Fusion (CAF) module is introduced at the intermediate semantic stage (E3) to perform adaptive feature alignment and fusion, thereby enhancing discrimination of complex tissue structures and small-volume nodules.
3. A Detail Context Fusion (DCF) based decoder is designed to replace conventional double-convolution units. Through a dual-branch architecture, the DCF block jointly enhances local detail representation and contextual semantic modeling, resulting in improved boundary delineation and structural integrity of the segmentation results.

## 2 Related work

In the early development of deep learning–based medical image segmentation, convolutional neural network (CNN)–based approaches played a dominant role. Long et al. (17) introduced the Fully Convolutional Network (FCN), which enabled end-to-end pixel-wise prediction and laid the foundation for modern semantic segmentation. To address spatial information loss caused by downsampling, SegNet was proposed by Badrinarayanan et al. (18), employing an encoder–decoder architecture with pooling index–guided upsampling. Although these early frameworks advanced dense prediction, achieving a balance between fine-grained detail preservation and high-level semantic representation remained challenging.

In the medical imaging domain, particularly for lung nodule segmentation, the U-Net architecture has become a canonical framework due to its symmetric encoder–decoder structure and skip connections, which effectively integrate low-level spatial details with deep semantic features. Building upon this design, DMC-UNet was proposed by Fan et al. (19) to enhance local texture modeling through multi-scale convolutional feature aggregation. Subsequently, Wu et al. (20) introduced RAD-UNet, incorporating attention mechanisms to improve feature discrimination in complex lung CT backgrounds. A residual and densely supervised variant, termed ResDSDA U-Net, was later developed by Ji et al. (21) to strengthen semantic representation and gradient propagation. More recently, SAtUNet was presented by Selvadass et al. (22), in which serial atrous convolutions were employed to expand the receptive field and improve multi-scale context modeling. In contrast, SaraNet was introduced by Wang et al. (23), leveraging a reverse attention mechanism to emphasize boundary-sensitive regions and enhance segmentation accuracy for small lung nodules. Despite these improvements, CNN-based methods remain inherently constrained by their limited receptive fields, which restrict effective modeling of long-range dependencies and global semantic context, particularly for small and adherent nodules in CT images. In addition to architecture-specific improvements, adaptive segmentation frameworks have also demonstrated strong performance in medical image analysis. Isensee et al. (24) proposed nnUNet, which automatically configures preprocessing, network architecture, training strategies, and postprocessing according to dataset characteristics, achieving strong robustness and generalization capability across diverse biomedical segmentation tasks.

To overcome these limitations, Transformer-based architectures have attracted increasing attention owing to their powerful self-attention mechanisms. The Vision Transformer (ViT) was proposed by Dosovitskiy et al. (25), demonstrating that self-attention can effectively capture global contextual relationships. Subsequently, Liu et al. (26) developed the Swin Transformer, which employs a hierarchical structure with shifted windows to improve computational efficiency while preserving global modeling capability. Building on these advances, Swin-UNet was introduced by Cao et al. (27) as a pure Transformer-based segmentation framework capable of modeling long-range dependencies and maintaining global structural consistency. However, the lack of inductive bias for local texture modeling in Transformer-based architectures often limits precise delineation of small anatomical structures such as lung nodules.

To jointly leverage the complementary strengths of CNNs and Transformers, hybrid CNN–Transformer architectures have emerged as an effective solution. Chen et al. (28) proposed TransUNet, embedding Transformer modules within the U-Net framework to enhance global contextual understanding. In contrast, UCTransNet was introduced by Wang et al. (29), in which skip connections are redesigned using channel-wise Transformer-based attention to alleviate semantic inconsistency between encoder and decoder representations. For lung nodule segmentation, Raza et al. (30) developed Trans RCED-UNet3+, incorporating Transformer-assisted semantic enhancement into a CNN-dominated architecture. Boundary-aware feature interaction was further emphasized in DPBET, which was proposed by Wang et al. (31) to strengthen nodule contour representation through a dual-path design. More recently, studies have continued to refine hybrid designs by focusing on spatial sensitivity and structural preservation. EDTNet was presented by Yadav et al. (32), integrating spatial-aware attention mechanisms to improve fine-grained structure modeling, whereas Li et al. (33) proposed CTBP-Net, employing cross-Transformer interaction and a bidirectional pyramid structure to enhance multi-scale dependency modeling. Collectively, these studies demonstrate that both Transformer-based and hybrid CNN–Transformer architectures have shown strong potential for lung nodule CT segmentation, particularly in improving small-target detection and boundary refinement under complex background conditions.

Despite the substantial progress achieved, several challenges still remain, including effective cross-branch feature fusion, collaborative modeling across heterogeneous receptive fields, and fine-grained detail reconstruction during decoding. Motivated by these challenges, we propose DPCrossU-Net, a dual-branch parallel encoder–decoder framework that introduces a double-path encoding strategy and an adaptive cross-fusion mechanism to enable efficient collaboration between local detail features and global semantic representations, thereby improving segmentation accuracy and robustness in complex CT imaging scenarios.

## 3 Methods

**Figure 1:**
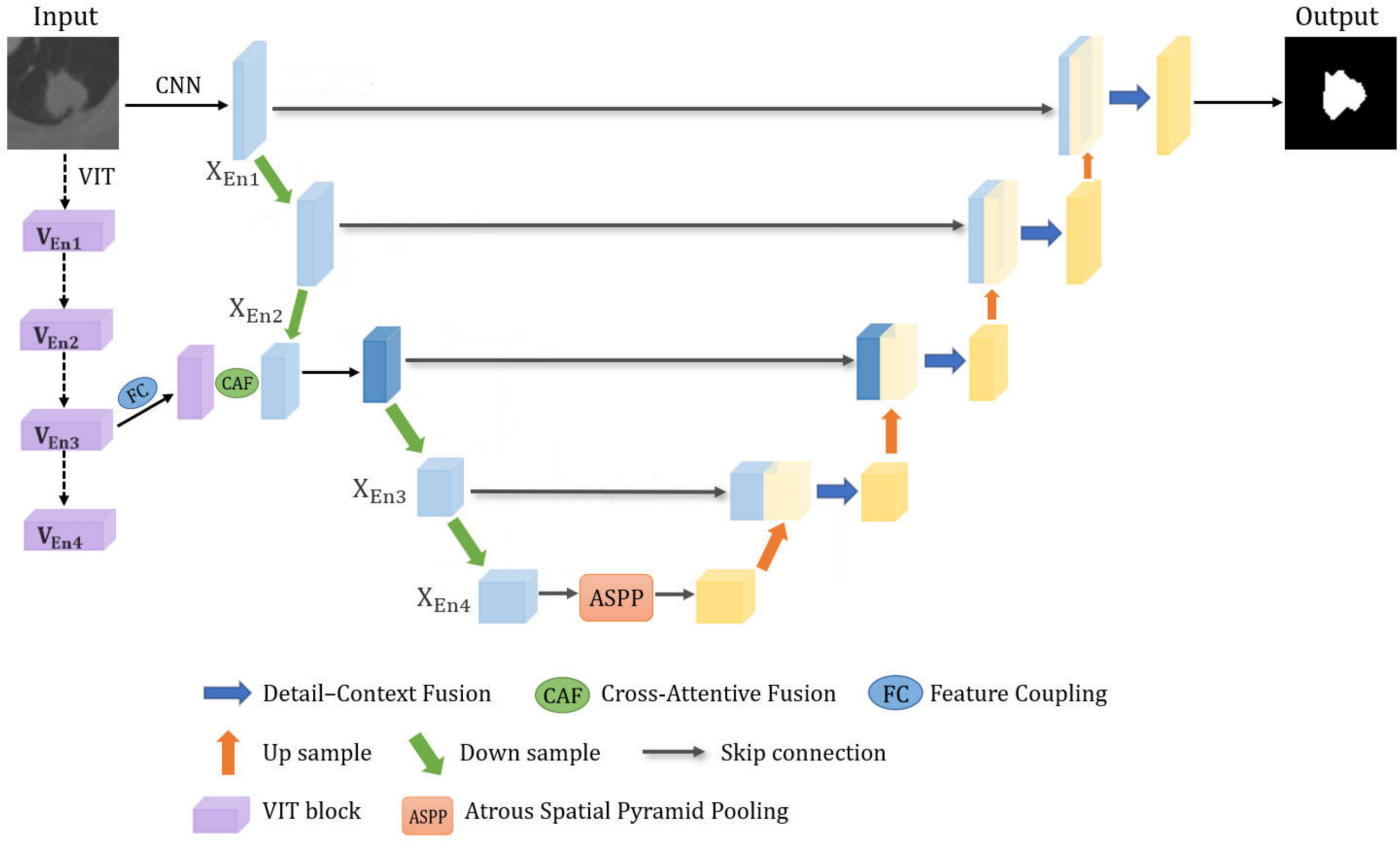
Illustration of the proposed DPCrossU-Net structure.

We propose a lung nodule segmentation framework, termed DPCrossU-Net, which integrates multi-scale convolutional operations with Transformer-based representations within a unified encoder–decoder architecture. The primary objective of this framework is to accurately segment lung nodules that are typically small in size, exhibit low contrast, and present high morphological variability in CT images. As illustrated in **Figure 1**, DPCrossU-Net adopts a U-shaped architecture with a dual-branch parallel design in the encoding stage, where a convolutional neural network (CNN) branch and a Transformer branch operate concurrently to capture complementary feature representations. Specifically, the CNN branch focuses on extracting fine-grained spatial details and boundary-related features, while the Transformer branch leverages self-attention mechanisms to model long-range dependencies and global semantic context.

To facilitate effective interaction between these heterogeneous feature representations, a Cross-Attention Fusion (CAF) module is introduced at the intermediate semantic stage (E3 layer), enabling dynamic feature alignment and adaptive cross-branch integration. This design alleviates feature inconsistency arising from differences in receptive fields and semantic hierarchies between the two branches. At the bottleneck layer, an Atrous Spatial Pyramid Pooling (ASPP) (34) module is employed to aggregate contextual information across multiple receptive fields, thereby enhancing the representation of structures at different scales, particularly small lung nodules. In the decoding stage, traditional double-convolution units are replaced with a dual-branch Detail Context Fusion (DCF) block. By jointly reconstructing shallow spatial details and deep semantic information through a multi-level feature refinement strategy, the decoder effectively improves boundary delineation and the recovery of fine-grained anatomical structures without increasing architectural complexity.

### 3.1 Dual-branch parallel encoder

To jointly model fine-grained local details and long-range global dependencies of lung nodules, a dual-branch parallel encoder is designed, comprising a convolutional branch and a Transformer branch that operate concurrently. The two branches progress synchronously throughout the encoding process while learning complementary feature representations: the convolutional branch emphasizes local structural details, whereas the Transformer branch focuses on capturing global semantic dependencies. To facilitate effective cross-branch interaction, identical spatial resolutions are maintained at corresponding encoding stages.

The convolutional branch follows a four-stage hierarchical feature extraction scheme derived from ResNet-50 (35), consisting of an initial convolutional stem followed by four backbone stages (E1–E4), corresponding to conv1, layer1, layer2, and layer3 in the original architecture. To ensure channel-level compatibility with the Transformer branch, each stage is equipped with a lightweight channel adaptation module composed of a 1×1 convolution followed by Batch Normalization. As a result, feature maps are projected to 128, 256, 512, and 512 channels at stages E1–E4, respectively, forming a hierarchy of local representations with progressively reduced spatial resolution and standardized channel dimensions.

The Transformer branch is constructed based on the ViT-Base/16 architecture and adopts a block-wise stage-output strategy. Input images are resized to 224×224 and processed through patch embedding and positional encoding before being fed into a stack of 12 Transformer encoder blocks. With increasing depth, token representations gradually evolve into globally contextualized semantic embeddings. To balance semantic expressiveness and spatial structure preservation, the output of the sixth encoder block is selected as the intermediate Transformer representation. After removing the class token, the remaining tokens are reshaped into a spatial feature map and projected to 512 channels using a 1×1 convolution, Batch Normalization, and GELU activation, enabling precise alignment with the convolutional features at stage E3.

Although spatial resolutions are kept consistent across branches, cross-branch interaction is not applied uniformly at all encoding stages. Shallow layers are primarily dominated by low-level texture statistics, whereas deeper Transformer features become highly abstract and may exhibit granularity mismatch with convolutional representations. In contrast, the intermediate E3 stage provides a favorable compromise between structural integrity and semantic stability. Accordingly, cross-branch alignment and adaptive fusion are exclusively performed at E3, resulting in a minimally invasive hybrid encoding strategy that mitigates semantic interference associated with multi-level fusion. Details of the proposed cross-branch alignment and fusion mechanism are described in the following section.

### 3.2 Feature coupling and cross-attentive fusion modules

Within the dual-branch encoder, the convolutional branch primarily captures fine-grained local texture details, whereas the Transformer branch focuses on modeling long-range semantic dependencies. Owing to inherent differences in spatial organization, representation format, and channel distribution, direct fusion of these heterogeneous features may result in semantic misalignment and information interference, thereby limiting the effectiveness of hybrid feature representations. To address this issue, a Feature Coupling (FC) module and a Cross-Attentive Fusion (CAF) module are introduced at the intermediate encoding stage, forming an align-first, interact-later fusion strategy that enables effective integration of local and global information across distinct representation spaces. The design of the proposed Feature Coupling and Cross-Attentive Fusion modules is inspired by existing feature fusion mechanisms reported in the literature (36), and is further adapted to accommodate the heterogeneous representations of convolutional and Transformer features in pulmonary nodule segmentation.

**Figure 2:**
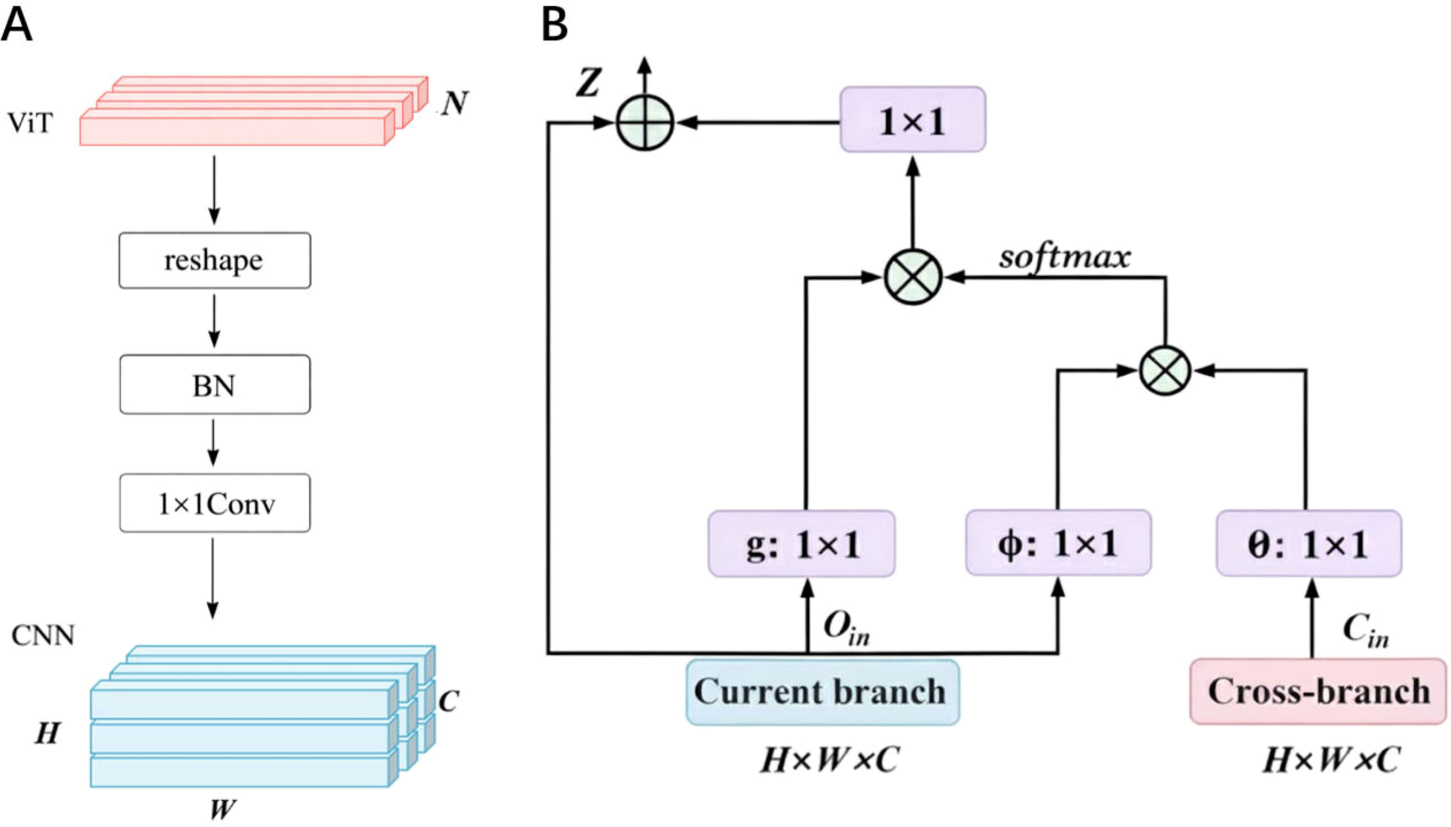
**(A)** Structural diagram of the feature coupling. **(B)** Cross-Attentive Fusion structure diagram.

The FC module is designed to resolve structural discrepancies between the two branches without introducing premature semantic interaction, as illustrated on the left side of **Figure 2**. Specifically, the Transformer output, originally represented as a sequence of tokens, is reshaped into a spatial feature map and normalized using batch normalization to stabilize cross-branch feature distributions. A 1×1 convolution is subsequently applied to project the Transformer features into the same channel dimensionality as the convolutional branch. Notably, this alignment process is performed unidirectionally from the Transformer branch to the convolutional branch, while the convolutional features remain unchanged. Through this operation, the FC module establishes a unified and compatible feature format, providing standardized inputs for subsequent attention-based fusion.

Following dimensional alignment, the CAF module adaptively integrates local texture cues and global semantic dependencies through a cross-branch attention mechanism, as illustrated on the right side of **Figure 2**. The convolutional features are projected into two complementary embeddings, denoted as g and *ϕ*, while the aligned Transformer features generate the semantic embedding *θ*. The embeddings *ϕ* and *θ* are flattened to compute spatial affinity, followed by a softmax operation to obtain the cross-branch attention matrix. This attention map is then applied to g, enabling the convolutional representation to be selectively enhanced by global contextual information. The resulting attention-enhanced feature is fused with the original convolutional feature via residual addition and further refined using a 1×1 convolution to produce the output feature *Z*. The overall fusion process is formally defined in Equation 1:

(1) $$F_{\text{CAF}}={\text{ConvBlock}}_{\text{CAF}}\left(\text{Concat}\left[{\text{Conv}}_{0}\left({\tilde{F}}_{\text{CNN}}\right)\cdot \text{softmax}\left(\frac{{\text{Conv}}_{1}{\left({\tilde{F}}_{\text{CNN}}\right)}^{⊤}{\text{Conv}}_{2}({\tilde{F}}_{\text{ViT}})}{\sqrt{C}}\right),\;F_{\text{CNN}}\right]\right)$$

By injecting long-range contextual cues from the Transformer branch into the stable convolutional backbone, the proposed fusion mechanism enhances the model’s ability to identify lung nodules with small size, blurred boundaries, or low contrast. Compared with simple feature summation or concatenation strategies, the attention-guided cross-branch fusion improves feature discriminability while maintaining a compact and coherent semantic representation. The fused feature is subsequently propagated as the sole input to deeper encoder layers, enabling more efficient and globally informed feature learning.

### 3.3 Atrous spatial pyramid pooling module

To enhance multi-scale contextual perception at the bottleneck stage, an Atrous Spatial Pyramid Pooling (ASPP) module is incorporated after the E4 layer of DPCrossU-Net. The primary role of this module is to aggregate contextual information across multiple receptive fields, thereby strengthening the semantic representation of lung nodules with varying sizes and indistinct boundaries before decoding. The ASPP module consists of multiple parallel branches, including a 1×1 convolution branch, three 3×3 atrous convolution branches with different dilation rates (1, 3, 5, and 7), and a global average pooling branch for capturing image-level contextual information. The outputs from all branches are concatenated along the channel dimension and subsequently fused through a residual connection followed by a 1×1 convolution, enabling effective cross-scale feature integration while preserving the original bottleneck representation. By jointly modeling local details, multi-scale context, and global semantic information without reducing spatial resolution, the ASPP module enriches the bottleneck features and provides more informative inputs for the subsequent decoder. This design contributes to more stable feature reconstruction and improved segmentation performance, particularly for lung nodules exhibiting large scale variations and ambiguous boundaries.

### 3.4 Detail context fusion block

After the encoder integrates multi-scale features and global semantic representations, the decoding stage is responsible for progressively restoring spatial details and reconstructing the boundary morphology of lung nodules. To effectively combine low-level structural information with high-level semantic cues during decoding, a dual-branch Detail Context Fusion (DCF) block is introduced. The DCF block is designed to enhance feature reconstruction through complementary convolutional pathways while maintaining a lightweight and efficient architecture.

**Figure 3:**
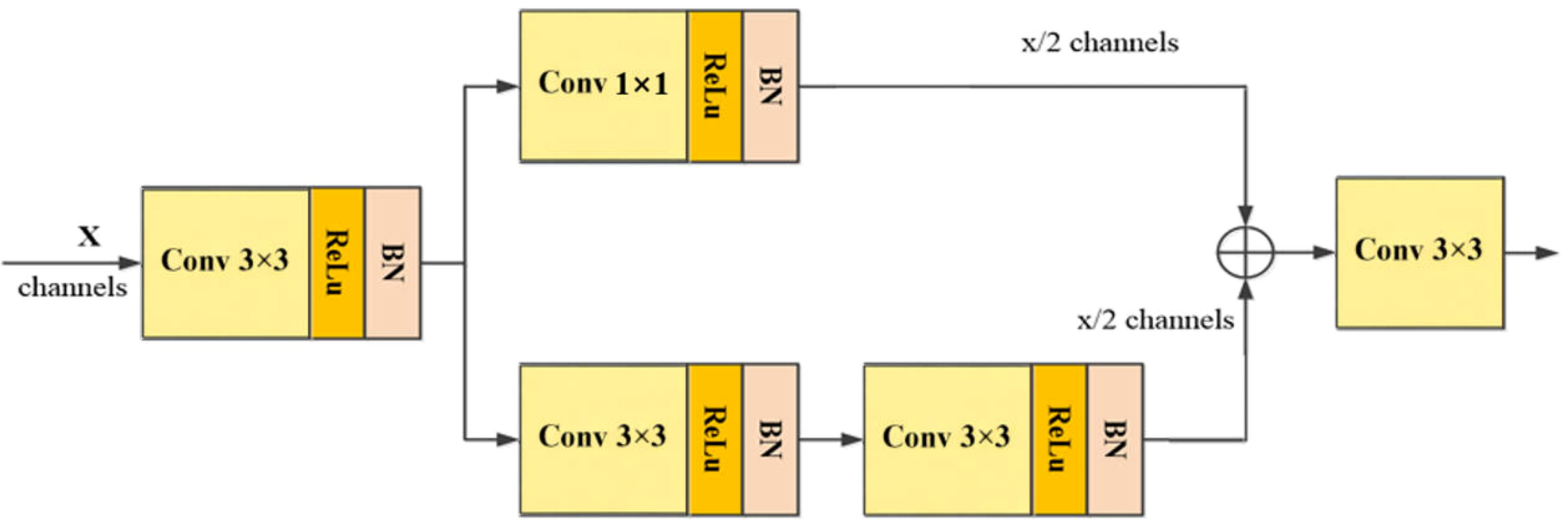
Architecture of the proposed detail context fusion block.

As illustrated in **Figure 3**, the input feature map is first processed by a 3×3 convolution followed by batch normalization (BN) and ReLU activation to unify feature representations before branching. The transformed features are then fed into two parallel convolutional pathways. The detail branch employs a single 1×1 convolution to emphasize high-resolution local structural information, with the output channels reduced to half of the input dimension after BN and ReLU, promoting compact detail representation. In contrast, the context branch utilizes two successive 3×3 convolutions to enlarge the effective receptive field and capture richer mid-scale and contextual semantics. Each convolution is followed by BN and ReLU to ensure stable optimization and non-linear feature modeling, and the output channels are similarly compressed to maintain dimensional consistency for subsequent fusion.

The outputs of the two branches are fused through channel-wise concatenation, followed by a 1×1 convolution to project the fused features into a unified representation, enabling effective integration of complementary local and global contextual information. The fused representation is further refined by an additional 3×3 convolution to improve spatial coherence during shape reconstruction and boundary recovery. A residual connection is incorporated to preserve the original semantic pathway and stabilize the decoding process. The overall formulation of the DCF block is summarized in Equation 2.

(2) $$F_{\text{DCF}}={\text{Conv}}_{3\times 3}\left(Concat\left[{\text{Conv}}_{1\times 1}\left({\text{Conv}}_{3\times 3}\left(X\right)\right),{\text{Conv}}_{3\times 3}\left({\text{Conv}}_{3\times 3}\left({\text{Conv}}_{3\times 3}\left(X\right)\right)\right)\right]\right)$$

By jointly modeling fine-grained details and contextual semantics through a dual-path design, the DCF block effectively addresses the small size, low contrast, and blurred boundaries commonly observed in lung nodules on CT images. This structured feature reconstruction strategy enables each decoding stage to generate discriminative spatial representations, providing reliable inputs for subsequent upsampling and contributing to more accurate and robust boundary delineation.

### 3.5 Loss function

To jointly optimize region-level overlap accuracy and pixel-wise classification performance in lung nodule segmentation, a weighted combination of Binary Cross-Entropy (BCE) loss and Dice Similarity Coefficient (DSC) loss is employed as the overall optimization objective. The BCE loss provides pixel-wise supervision on predicted probabilities, facilitating reliable discrimination between nodule and background regions, whereas the DSC loss directly optimizes the overlap between the predicted mask and the ground-truth annotation, making it particularly effective for small target structures and severe foreground–background imbalance.

By integrating these two complementary loss terms, the model achieves stable pixel-level classification while enhancing the representation of global nodule shape and boundary delineation, thereby improving convergence stability and segmentation accuracy. The formulations of the BCE loss, Dice loss, and their weighted combination are given in Equations 3–5. Here, σ(·) denotes the Sigmoid activation function, with *p_i_* = σ(*x_i_*); *ϵ* is introduced as a smoothing constant to prevent division by zero, and the weighting coefficient *w* is set to 0.5.

(3) $${\text{Loss}}_{\text{BCE}}=-\frac{1}{N}\sum_{i=1}^{N}\left[y_{i}\log\sigma \left(x_{i}\right)+\left(1-y_{i}\right)\log\left(1-\sigma \left(x_{i}\right)\right)\right]$$

(4) $$Loss_{Dice}=1-\frac{2\sum_{i}p_{i}y_{i}+\epsilon }{\sum_{i}p_{i}+\sum_{i}y_{i}+\epsilon }$$

(5) $$Loss=w\cdot Loss_{BCE}+\left(1-w\right)\cdot Loss_{Dice}$$

## 4 Experiments and discussion

### 4.1 Dataset

Experiments were conducted on the publicly available LIDC-IDRI dataset (37), which consists of spiral chest CT scans from 1,018 patients. Each scan was independently annotated by four experienced thoracic radiologists following the LIDC four-level annotation protocol, providing detailed delineation of lung nodule boundaries and attributes. To ensure annotation reliability, only nodules confirmed by at least two radiologists were retained, while ambiguous or highly inconsistent annotations were excluded.

After applying a minimum diameter threshold of 3 mm, a total of 6,959 valid nodule slices were obtained. For each nodule, the central slice together with one adjacent slice before and after was included to provide limited contextual information. Nodule locations were extracted from the official XML annotation files. For each nodule, a fixed-size region centered at the annotated centroid was cropped to a resolution of 64×64 pixels. This cropping strategy focuses the model on local lesion regions while reducing irrelevant background interference, which is particularly important for small pulmonary nodules. The dataset was randomly divided at the patient level into training, validation, and test sets with a ratio of 8:1:1, corresponding to 5,567, 695, and 697 slices, respectively. During training, online data augmentation techniques, including random rotation, translation, scaling, horizontal flipping, and intensity perturbation, were applied to enhance model generalization.

### 4.2 Experimental settings

All experiments were implemented using Python 3.10 and PyTorch 2.5 on a workstation equipped with an NVIDIA GeForce RTX 4060 GPU (16 GB VRAM). Automatic mixed precision (AMP) (38) was enabled to improve computational efficiency and reduce memory consumption.

The models were trained for 400 epochs with a batch size of 16 using the AdamW optimizer (39), with an initial learning rate of 1×10^-5^ and a weight decay of 0.001. A cosine annealing learning rate scheduler with a warm-up strategy during the first five epochs was adopted to accelerate convergence. Since the models converged within approximately 150 epochs, only the primary convergence stage is visualized in the training curves for clarity.

To maintain a consistent spatial resolution required by the network and facilitate stable optimization during training, all cropped patches were resized to 256×256 using bilinear interpolation, which preserves the overall morphological structure of lung nodules and is widely adopted in medical image segmentation studies. Moreover, the resizing operation was also necessary to satisfy the fixed input resolution requirements of the Transformer branch and maintain architectural consistency across different baseline models.

A fixed random seed was used throughout all experiments to ensure reproducibility. An early stopping strategy was employed, where training was terminated if the validation loss did not decrease for 30 consecutive epochs. The model with the highest Dice similarity coefficient (DSC) on the validation set was selected for final evaluation on the test set.

To improve the reliability of the reported results and reduce the influence of random initialization, all experiments were independently repeated five times using different random seeds. The final performance is reported as the mean value with standard deviation. To evaluate statistical significance, paired t-tests were performed on DSC scores obtained from multiple independent runs, with a p-value< 0.05 considered statistically significant.

### 4.3 Evaluation metrics

Segmentation performance was quantitatively evaluated using four widely adopted metrics: Dice Similarity Coefficient (DSC), Precision (PRE), Sensitivity (SEN), and Intersection over Union (IoU). Among them, DSC was used as the primary metric to measure the overlap between predicted segmentation results and ground-truth annotations. Precision and Sensitivity were employed to assess the accuracy and completeness of nodule detection, respectively, while IoU quantified the spatial consistency between predicted and reference regions.

After binarizing both predicted and ground-truth masks using a threshold of 0.5, the numbers of true positive (TP), false positive (FP), and false negative (FN) pixels were computed. The evaluation metrics were defined as Equations 6–9:

(6) $$\text{DSC}=\frac{2TP}{2TP+FP+FN}$$

(7) $$\text{PRE}=\frac{TP}{TP+FP}$$

(8) $$\text{SEN}=\frac{TP}{TP+FN}$$

(9) $$\text{IoU}=\frac{TP}{TP+FP+FN}$$

As this study focuses on a binary segmentation task, IoU is equivalent to the binary form of mean IoU (mIoU). All metrics range from 0 to 1, with higher values indicating better segmentation performance. Specifically, DSC and IoU primarily reflect regional overlap quality, whereas Precision and Sensitivity characterize the model’s specificity and sensitivity. The combined use of these metrics enables a comprehensive and objective assessment of segmentation performance.

### 4.4 Ablation studies

Ablation experiments were conducted to systematically assess the contribution of each key component in the proposed DPCrossU-Net, including the dual-branch parallel encoder with cross-branch fusion, the ASPP-based multi-scale context module, the DCF-based decoder, and the hybrid BCE–Dice loss optimization strategy. All ablation models were evaluated using identical data splits, training settings, and evaluation metrics, with the complete DPCrossU-Net serving as the reference baseline to ensure fair and consistent comparisons.

#### 4.4.1 Effectiveness of the dual-branch parallel encoder

This subsection investigates the effectiveness of the proposed CNN–ViT dual-branch encoder in modeling multi-scale lung nodule features and further analyzes the influence of different cross-branch interaction strategies and fusion locations.

To this end, several baseline architectures were constructed for comparison. A single-branch convolutional model based on the ResNet-50 U-Net architecture (Res-UNet) was employed to capture local texture patterns and boundary details, while a single-branch Transformer-based model (ViT-UNet) was utilized to model long-range contextual dependencies and global semantic information. In addition, a dual-branch parallel architecture was designed by retaining both ResNet-50 and ViT encoding pathways without explicit cross-branch interaction, where features from the two branches were fused only at the bottleneck stage through feature concatenation.

Compared with the single-branch baselines, the dual-branch parallel architecture achieved improved segmentation performance, demonstrating the complementary characteristics of convolutional local representations and Transformer-based global contextual modeling. However, the absence of explicit feature interaction between the two branches limited further performance improvement.

To address this issue, the proposed Cross-Attentive Fusion (CAF) module was introduced at different encoder stages to facilitate adaptive cross-branch feature interaction. As shown in **Table 1**, integrating CAF at the intermediate encoder stage (E3) achieved the best segmentation performance and significantly outperformed the dual-branch model without fusion. In contrast, applying CAF at deeper stages (E4) or simultaneously at both E3 and E4 did not produce further improvement.

**Table 1:** Dual-branch parallel encoder and fusion strategy.

Model configuration	Cross-branch interaction strategy	DSC(%)	IoU(%)	PRE(%)	SEN (%)
Res-UNet	None	85.11	74.92	86.80	86.47
ViT-UNet	None	85.08	74.83	86.71	86.34
Dual-Parallel	None	85.32	75.51	87.08	86.54
DP+CAF (E3-E4)	CAF (E3+E4)	85.18	75.23	86.87	86.53
DP+CAF (E4)	CAF (E4)	85.22	75.32	87.01	86.55
DP+CAF (E3)	CAF (E3)	85.43	75.84	87.16	86.67

This phenomenon can be explained by the hierarchical representation characteristics of the encoder. Shallow-stage features mainly contain low-level texture and edge information with limited semantic abstraction, resulting in insufficient compatibility between CNN and Transformer representations. In contrast, deep-stage features contain highly abstract semantic information but suffer from substantial spatial resolution loss, which weakens precise boundary localization for small lung nodules.

The intermediate encoder stage (E3) provides a more suitable balance between semantic representation and spatial detail preservation. At this stage, convolutional features retain sufficient local structural information, while Transformer features already capture meaningful global contextual dependencies. Therefore, feature complementarity and interaction compatibility between the two branches become more favorable for adaptive fusion.

By introducing CAF at the E3 stage, the proposed model effectively integrates locally enhanced convolutional features with globally informed Transformer representations, generating more discriminative feature embeddings for subsequent encoding and decoding stages. Overall, these results demonstrate the effectiveness of the CNN–ViT dual-branch architecture and highlight the importance of intermediate-stage cross-branch interaction for accurate lung nodule segmentation.

#### 4.4.2 Effectiveness of the ASPP-based module

This subsection investigates the effect of atrous convolution receptive field configurations and residual enhancement on pulmonary nodule segmentation performance. Under a fixed ASPP architecture, different dilation rate strategies were evaluated while keeping all other components unchanged. The default configuration, denoted as A_0_, adopts linearly increasing dilation rates of (1, 3, 5, 7), which provide continuous receptive field coverage from local texture patterns to mid-scale semantic contexts with limited redundancy. For comparison, three alternative dilation strategies were considered: a coprime configuration (1, 2, 5, 9) to enhance cross-scale complementarity, an exponential expansion (1, 2, 4, 8) as a classical reference, and a densely distributed small-scale setting (1–4) to emphasize fine structural responses. Experimental results show that the linear configuration A_0_ consistently yields more stable and better overall performance, whereas the alternative strategies fail to provide additional benefits. This observation suggests that linear dilation growth is better suited to the structural characteristics of small, low-contrast pulmonary nodules commonly encountered in CT images.

Based on this finding, lightweight residual connections were further incorporated into the ASPP module to strengthen multi-branch feature consistency and improve contextual representation at the bottleneck. The residual enhancement leads to more robust multi-scale feature fusion without increasing architectural complexity. Consequently, the final model adopts the linear dilation configuration (1, 3, 5, 7) with residual enhancement as the ASPP design.

#### 4.4.3 Effectiveness of the DCF block and hierarchical configuration

This subsection investigates the effectiveness of the proposed Detail Context Fusion (DCF) block by analyzing its deployment at different decoder levels and its influence on segmentation performance. To ensure fair comparison, all configurations were evaluated using identical training strategies and parameter settings. The examined configurations include single-level deployment, partial multi-level combinations, and full multi-level deployment across the decoder, with the Dice Similarity Coefficient (DSC) used as the primary evaluation metric. Quantitative results are summarized in **Table 2**.

**Table 2:** DCF Block hierarchical configuration.

Configuration	Enabled decoder levels	DSC(%)	IoU(%)	PRE(%)	SEN(%)
Baseline	None	85.43	75.84	87.16	86.67
D4 only	D4	85.67	76.16	87.23	86.98
D3 only	D3	85.31	75.59	86.93	86.61
D3+D4	D3, D4	85.24	75.49	86.72	86.42
D2+D3	D2, D3	85.42	75.68	86.97	86.65
D1-D4	All layers	85.89	76.42	87.48	87.11

The baseline model without the DCF block achieves a DSC of 85.43%. Introducing the DCF block only at the deepest decoder stage (D4) improves the DSC to 85.67%, indicating that high-level semantic features provide effective contextual constraints for feature reconstruction. In contrast, deploying the DCF block solely at the intermediate decoder level (D3) yields only marginal improvement, suggesting that mid-level features alone are insufficient to fully exploit the block’s representational capacity. Moreover, partial multi-level combinations do not lead to additive performance gains; in some cases, such as the D3+D4 configuration, performance even degrades compared to using D4 alone, implying limited cross-scale coordination under localized deployment.

The best overall performance is achieved when the DCF block is deployed across all decoder levels (D1–D4), resulting in a DSC of 85.89%. This improvement demonstrates the advantage of hierarchical collaborative enhancement during decoding. Specifically, deep decoder features provide stable global semantic guidance, while shallower features progressively refine boundary details and fine-grained structures. Joint optimization across all decoder stages enables more consistent and reliable feature reconstruction. Under this full deployment configuration, auxiliary metrics including IoU, Precision, and Sensitivity also reach their optimal values, further confirming its effectiveness in both regional consistency and boundary delineation. Accordingly, the D1–D4 full deployment strategy is adopted for the final model.

#### 4.4.4 Effectiveness of the loss function

This subsection investigates the influence of different loss function configurations on pulmonary nodule segmentation performance. Since lung nodules usually occupy only a small portion of CT images and exhibit severe foreground-background imbalance, appropriate loss design is essential for stable optimization and accurate boundary delineation. To ensure fair comparison, all experiments were conducted using the complete DPCrossU-Net architecture while only varying the loss function configuration.

Specifically, five loss settings were evaluated, including BCE loss, Dice loss, and different weighted combinations of BCE and Dice losses. Quantitative results are summarized in **Table 3**.

**Table 3:** Ablation study of different loss function configurations.

Loss function	DSC (%)	IoU (%)	PRE (%)	SEN (%)
BCE Loss	85.08	75.91	86.72	86.31
Dice Loss	85.24	76.08	86.93	86.48
0.7 BCE + 0.3 Dice	85.52	76.21	87.14	86.63
0.3 BCE + 0.7 Dice	85.61	76.33	87.29	86.92
0.5 BCE + 0.5 Dice	85.89	76.42	87.48	87.11

Experimental results demonstrate that the combined loss functions consistently outperform single loss functions. BCE loss focuses primarily on pixel-wise classification accuracy but is sensitive to class imbalance, whereas Dice loss directly optimizes regional overlap but may lead to unstable convergence in difficult samples. By combining the two objectives, the model achieves improved balance between local pixel supervision and global structural consistency.

Among all configurations, the equal-weight combination of BCE and Dice loss achieves the best overall performance, yielding a DSC of 85.89% and an IoU of 76.42%. This result indicates that balanced optimization of pixel-level discrimination and overlap consistency is particularly beneficial for pulmonary nodule segmentation, especially in challenging cases involving small nodules and ambiguous boundaries. Therefore, the equal-weight BCE–Dice loss is adopted in the final model.

#### 4.4.5 Overall performance

The ablation studies collectively demonstrate that the proposed segmentation framework benefits from the complementary collaboration of its encoder, bottleneck, decoder, and optimization components. The dual-branch CNN–ViT encoder enhances both local detail representation and global semantic modeling, while adaptive cross-branch fusion facilitates effective multi-scale feature integration and improves robustness across diverse nodule appearances. Contextual modeling is further strengthened by the ASPP module at the bottleneck, which enriches semantic representations for subsequent decoding. At the decoding stage, the hierarchical deployment of the DCF block enables progressive refinement of spatial details and boundary structures. In addition, the hybrid BCE–Dice loss further improves optimization stability and segmentation consistency by jointly considering pixel-wise classification accuracy and regional overlap constraints.

Experimental results from the ablation studies indicate that each proposed component contributes positively to the overall segmentation performance. The combination of the dual-branch encoder, adaptive feature fusion, ASPP-based contextual enhancement, hierarchical DCF decoding strategy, and hybrid loss optimization achieves the best overall quantitative performance across DSC, IoU, Precision, and Sensitivity metrics.

Together, these components form a synergistic segmentation framework that achieves stable and accurate pulmonary nodule delineation across diverse clinical scenarios, particularly in challenging cases involving small, irregular, low-contrast, and juxta-pleural nodules.

### 4.5 Quantitative comparison with representative segmentation methods

To comprehensively evaluate the effectiveness of the proposed DPCrossU-Net for lung nodule segmentation, comparative experiments were conducted against representative CNN-based and Transformer-based segmentation frameworks, including U-Net, SaraNet, nnUNet, TransUNet, and Swin-UNet. All models were trained and evaluated under identical experimental settings, including dataset partitioning, input resolution, data augmentation strategies, optimization settings, and training epochs, to ensure fair and consistent comparison. Segmentation performance was evaluated using four widely adopted metrics, including Dice Similarity Coefficient (DSC), Intersection over Union (IoU), Precision (PRE), and Sensitivity (SEN).

To further assess model robustness and reduce the influence of random initialization, all experiments were independently repeated five times using different random seeds, and the mean and standard deviation of each evaluation metric were reported.

Quantitative comparison results are summarized in **Table 4**. Conventional CNN-based methods, including U-Net and SaraNet, achieve relatively stable segmentation performance but remain limited in modeling long-range contextual dependencies, particularly for small or low-contrast lung nodules. Although SaraNet improves boundary-aware feature representation through reverse attention mechanisms, segmentation inaccuracies still occur in regions with blurred boundaries and complex anatomical structures.

**Table 4:** Quantitative comparison of segmentation performance for different methods on the LIDC-IDRI dataset.

Model	DSC (%)	IoU (%)	PRE (%)	SEN (%)	Params (M)	FLOPs (G)
U-Net	84.43 ± 0.42	74.71 ± 0.39	86.29 ± 0.35	86.30 ± 0.41	22.3	38.6
SaraNet	84.87 ± 0.37	74.89 ± 0.33	86.38 ± 0.31	86.45 ± 0.36	36.5	51.3
nnUNet	84.98 ± 0.34	75.05 ± 0.30	86.45 ± 0.28	86.52 ± 0.33	38.7	58.2
TransUNet	85.19 ± 0.29	75.28 ± 0.26	86.78 ± 0.24	86.68 ± 0.27	89.1	89.5
Swin-UNet	85.01 ± 0.31	75.09 ± 0.28	86.54 ± 0.26	86.55 ± 0.29	51.5	66.2
DPCrossU-Net	85.89 ± 0.23	76.42 ± 0.21	87.48 ± 0.19	87.11 ± 0.22	59.4	72.6

The self-configuring nnUNet framework demonstrates improved segmentation capability owing to its adaptive preprocessing and training strategies, achieving more robust performance than conventional CNN-based architectures. Transformer-based models, including TransUNet and Swin-UNet, further improve global contextual modeling and achieve higher DSC and IoU values compared with pure CNN-based methods. However, their ability to preserve fine-grained boundary information remains constrained because accurate local structural modeling still relies heavily on convolutional operations.

Compared with all baseline methods, the proposed DPCrossU-Net achieves the best overall segmentation performance, with DSC, IoU, PRE, and SEN values of 85.89%, 76.42%, 87.48%, and 87.11%, respectively. The superior performance can be attributed to the dual-branch parallel CNN–ViT encoder, adaptive cross-branch feature fusion mechanism, ASPP-based multi-scale contextual modeling, and progressive feature refinement in the decoder stage.

In addition to segmentation accuracy, computational complexity was further evaluated by comparing the number of model parameters and FLOPs. As shown in **Table 4**, although the proposed DPCrossU-Net introduces additional computational cost compared with conventional CNN-based models, it still maintains lower model complexity than TransUNet while achieving superior segmentation performance. These results demonstrate that the proposed architecture achieves a favorable balance between segmentation accuracy and computational efficiency.

Furthermore, paired t-tests were conducted between DPCrossU-Net and representative Transformer-based baseline methods, including TransUNet and Swin-UNet, using the DSC and IoU evaluation metrics. As shown in **Table 5**, the proposed method achieves statistically significant improvements over both baseline models (p< 0.01) across all evaluated metrics, demonstrating that the observed performance gains are reliable rather than caused by random fluctuations.

**Table 5:** Statistical significance analysis of DSC scores between DPCrossU-Net and representative Transformer-based methods.

Comparison	Metric	t-value	p-value	Significance
TransUNet vs DPCrossU-Net	DSC	-3.17	0.0018	Significant
TransUNet vs DPCrossU-Net	IoU	-3.42	0.0013	Significant
Swin-UNet vs DPCrossU-Net	DSC	-3.39	0.0015	Significant
Swin-UNet vs DPCrossU-Net	IoU	-3.68	0.0011	Significant

**Figure 4:**
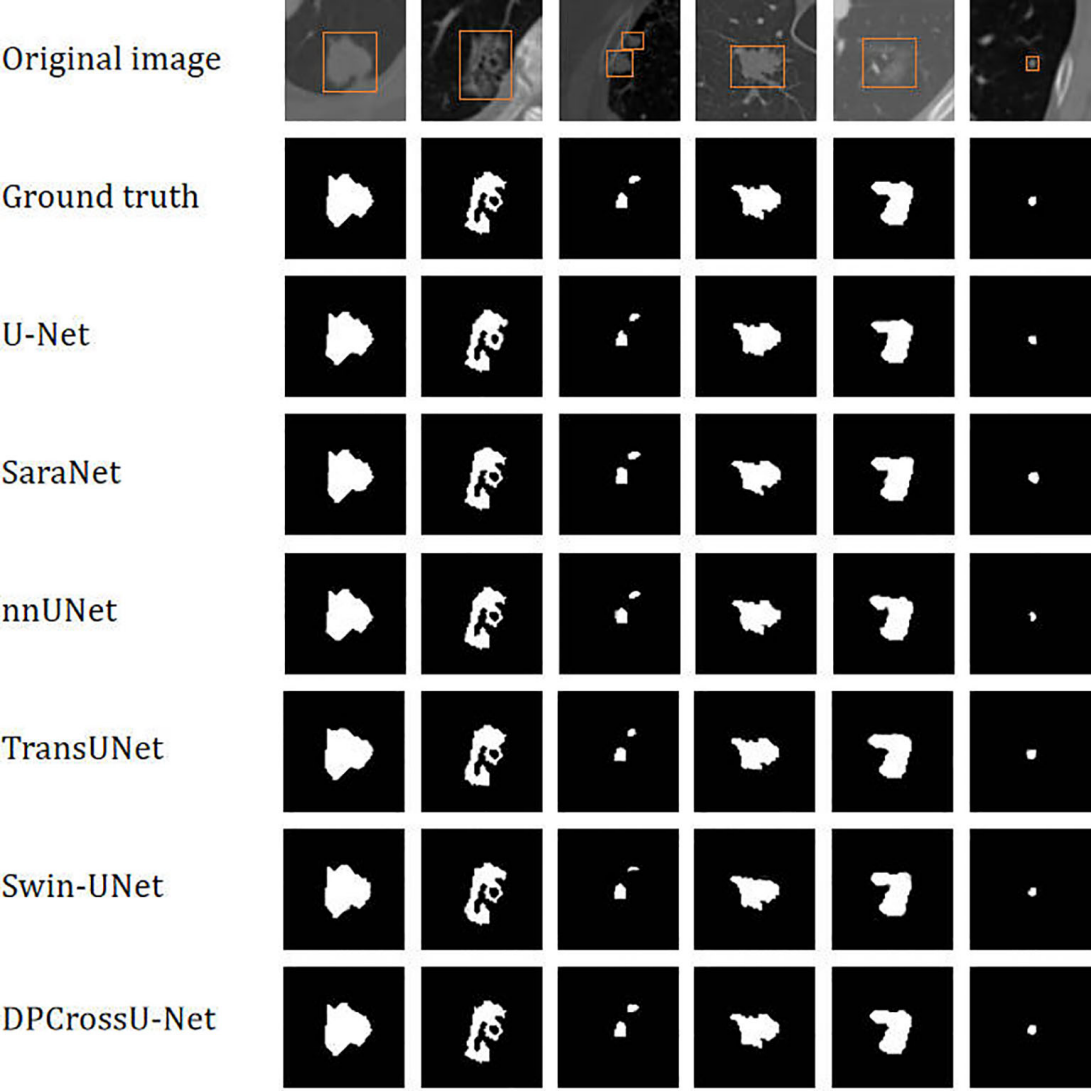
A visual comparison of the segmentation results. From top to bottom: original image, ground truth of nodules, segmentation results of various methods.

**Figure 5:**
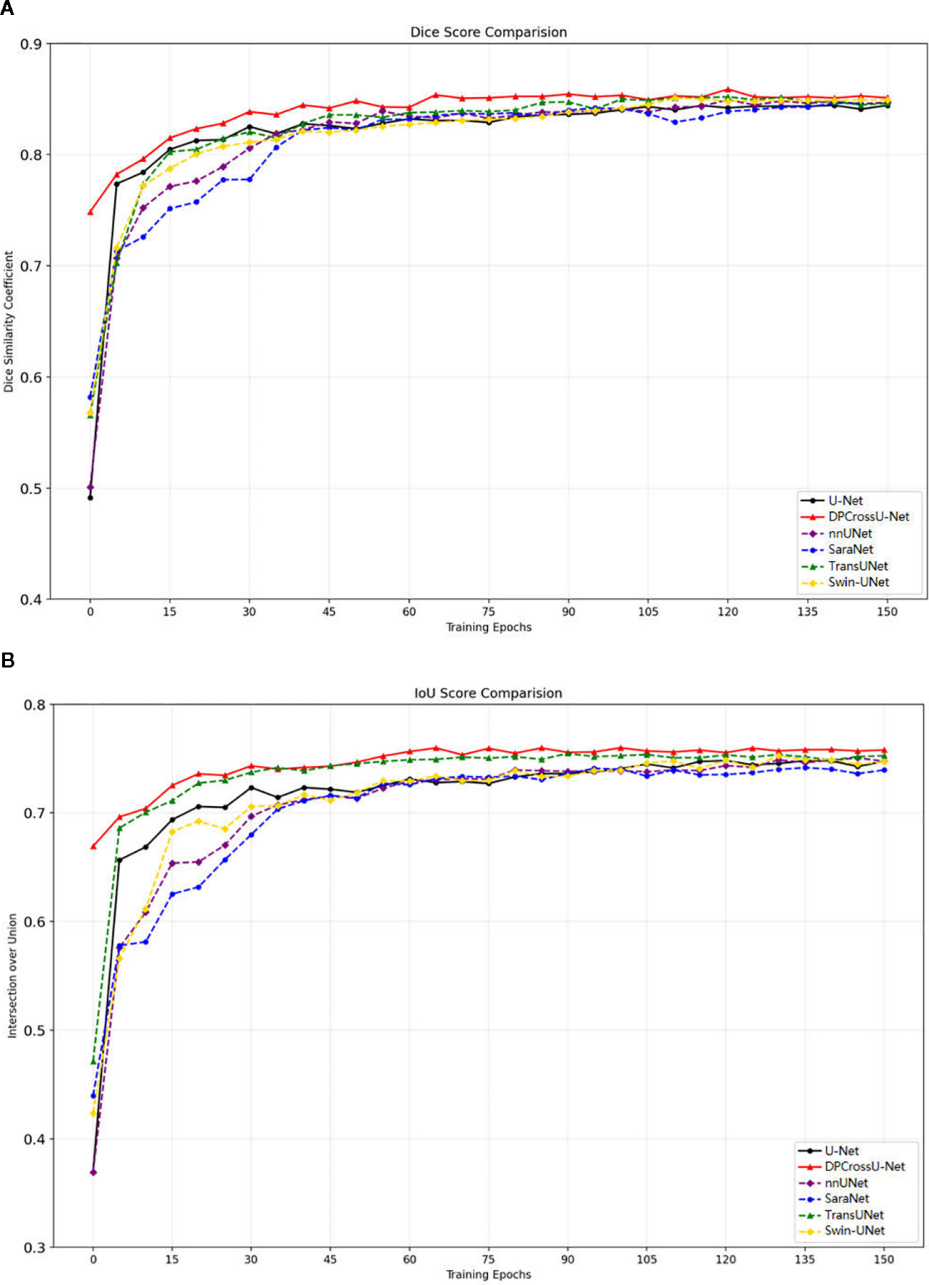
Quantitative performance comparison during training: **(A)** Dice score comparison and **(B)** IoU score comparison.

Overall, the proposed DPCrossU-Net consistently outperforms all baseline methods and demonstrates superior robustness in challenging scenarios involving small nodules, weak boundaries, and complex anatomical structures. Qualitative segmentation comparisons for different methods are presented in **Figure 4**, while quantitative performance trends during training are illustrated in **Figure 5**, further validating the effectiveness and robustness of the proposed approach.

### 4.6 Discussion

The experimental results demonstrate that the proposed DPCrossU-Net achieves consistently improved segmentation performance on the LIDC-IDRI dataset. These improvements are mainly attributed to the effective integration of local detail modeling and global contextual representation within the dual-branch CNN–Transformer architecture.

The proposed CAF module plays an important role in bridging the representational gap between convolutional and Transformer features. By enabling adaptive cross-attention between the two branches, the model facilitates effective information exchange between local texture-sensitive representations and global dependency-aware semantic features, resulting in more discriminative feature embeddings.

The superior performance observed at the intermediate encoder stage (E3) further highlights the importance of balancing semantic abstraction and spatial detail preservation during cross-branch interaction. Shallow-stage features mainly contain low-level texture information with insufficient semantic representation, whereas deep-stage features become spatially coarse despite stronger semantic abstraction. Intermediate-stage features provide more favorable conditions for effective CNN–Transformer feature interaction and adaptive fusion.

The ASPP module further improves multi-scale contextual representation by enlarging the receptive field and capturing features at different spatial scales, which is particularly beneficial for segmenting small and low-contrast lung nodules. In addition, the hierarchical deployment of the DCF block progressively refines boundary information during decoding and improves structural consistency in the final segmentation outputs.

To further evaluate model reliability, multiple independent experiments with different random seeds were conducted, and statistical significance analyses were performed. Experimental results demonstrate that the performance improvements achieved by DPCrossU-Net are statistically significant rather than caused by random fluctuations, indicating favorable robustness and reproducibility.

In addition, computational complexity analysis shows that introducing the Transformer branch inevitably increases model parameters and FLOPs compared with conventional CNN-based architectures. Nevertheless, the proposed model still maintains a reasonable trade-off between segmentation accuracy and computational cost when compared with other Transformer-based segmentation methods.

## 5 Conclusion and future work

This study proposes DPCrossU-Net, a hybrid CNN–Transformer framework designed to address key challenges in lung nodule segmentation, including scale variation, indistinct boundaries, and low contrast in CT images. By integrating a dual-branch parallel encoder with adaptive cross-feature fusion, atrous convolution–based contextual enhancement, and a detail-aware decoding strategy, the proposed method achieves superior segmentation accuracy and robustness compared with representative state-of-the-art approaches across multiple evaluation metrics. Notably, consistent performance improvements are observed in challenging cases involving small-volume, low-contrast, and juxta-pleural nodules, demonstrating the effectiveness of jointly modeling local details and global semantic context.

Despite these encouraging results, several directions remain for future investigation. First, the current framework operates on two-dimensional slice-level inputs. Extending the proposed architecture to three-dimensional volumetric representations may further improve spatial continuity modeling and inter-slice dependency capture. Second, although the Transformer branch enhances global contextual perception, it introduces additional computational and memory overhead. Future work will explore lightweight attention mechanisms, sparse modeling strategies, and network optimization techniques to improve efficiency and scalability.

In addition, the current study evaluates the proposed framework only on the publicly available LIDC-IDRI dataset. Although the experimental results demonstrate strong segmentation performance, the lack of external multi-center validation may limit the assessment of model generalizability under heterogeneous clinical conditions. Future work will therefore include cross-dataset evaluation and external validation on multi-center CT datasets acquired from different scanners and imaging protocols.

Furthermore, clinical CT data often exhibit substantial variability across scanners and acquisition protocols. Investigating domain generalization and cross-center learning strategies may help enhance robustness under heterogeneous data distributions. Future work will also further investigate explainability and feature similarity analysis for cross-branch interaction mechanisms, which may provide deeper insight into the complementary behavior between CNN and Transformer representations. In addition, extending the segmentation framework toward joint learning with detection or classification tasks, or incorporating structural priors from large-scale medical foundation models, represents a promising direction for further improving clinical applicability.

## Data Availability

Publicly available datasets were analyzed in this study. These data can be found in The Cancer Imaging Archive (TCIA), LIDC-IDRI collection: https://www.cancerimagingarchive.net/collection/lidc-idri/.
