# Correction: DPCrossU-Net: a dual-branch parallel CNN–Transformer network for lung nodule segmentation

**DOI:** 10.3389/fonc.2026.1905124

**Published:** 2026-07-09

**Authors:** Xiya Guan, Wen Zhu, Fangxiang Wu

**Affiliations:** 1 School of Mathematics and Statistics, Hainan Normal University, Haikou, China; 2 School of Mathematics and Systems Science, Guangdong Polytechnic Normal University, Guangzhou, China; 3 Division of Biomedical Engineering and Department of Mechanical Engineering, University of Saskatchewan, Saskatoon, SK, Canada

**Keywords:** CNN–Transformer hybrid model, dual-branch architecture, feature fusion, LIDC-IDRI, lung nodule segmentation

There was a mistake in **Table 2** as published. In the second row “Baseline”, “None”, the Precision (Pre, %) value was incorrectly reported as “87.06”. The correct value is “87.16”. The corrected **Table 2** appears below.

**Table 2:** DCF Block hierarchical configuration.

Configuration	Enabled decoder levels	DSC(%)	IoU(%)	PRE(%)	SEN(%)
Baseline	None	85.43	75.84	87.16	86.67
D4 only	D4	85.67	76.16	87.23	86.98
D3 only	D3	85.31	75.59	86.93	86.61
D3+D4	D3, D4	85.24	75.49	86.72	86.42
D2+D3	D2, D3	85.42	75.68	86.97	86.65
D1-D4	All layers	85.89	76.42	87.48	87.11

The IoU value in the first sentence of the last paragraph of Section 4.4.3 “*Effectiveness of the DCF block and hierarchical configuration*” was incorrectly reported as “77.94%” instead of “76.42%”.

A correction has been made to the section 4 **Experiments and discussion**, 4.4.3 “*Effectiveness of the DCF block and hierarchical configuration*”, last paragraph:

“Among all configurations, the equal-weight combination of BCE and Dice loss achieves the best overall performance, yielding a DSC of 85.89% and an IoU of 76.42%. This result indicates that balanced optimization of pixel-level discrimination and overlap consistency is particularly beneficial for pulmonary nodule segmentation, especially in challenging cases involving small nodules and ambiguous boundaries. Therefore, the equal-weight BCE–Dice loss is adopted in the final model.”

The original version of this article has been updated.

